# Dyspnea, a high-risk symptom in patients suspected of myocardial infarction in the ambulance? A population-based follow-up study

**DOI:** 10.1186/s13049-016-0204-9

**Published:** 2016-02-12

**Authors:** Morten Thingemann Bøtker, Carsten Stengaard, Mikkel Strømgaard Andersen, Hanne Maare Søndergaard, Karen Kaae Dodt, Troels Niemann, Hans Kirkegaard, Erika Frischknecht Christensen, Christian Juhl Terkelsen

**Affiliations:** Department for Research and Development, Prehospital Emergency Medical Services, Central Denmark Region, Olof Palmes Allé 32, 1, 8200 Aarhus, N Denmark; Department of Cardiology B, Aarhus University Hospital, 8200 Aarhus, N Denmark; Department of Cardiology, Viborg Regional Hospital, 8800 Viborg, Denmark; Department of Cardiology, Horsens Regional Hospital, 8700 Horsens, Denmark; Department of Cardiology, Herning Regional Hospital, 7400 Herning, Denmark; Research Center for Emergency Medicine, Aarhus University Hospital, 8000 Aarhus, C Denmark; Deparment of Clinical Medicine, Deparment of Clinical Medicine, 9100 Aalborg, Denmark

**Keywords:** Dyspnea, Myocardial infarction, Prehospital emergency medical services, Mortality

## Abstract

**Background:**

Systematic management of patients suffering high-risk symptoms is essential in emergency medical services. Patients with chest pain receive algorithm-based work-up and treatment. Though dyspnea is recognized as an independent predictor of mortality, no generally accepted prehospital treatment algorithm exists and this may affect outcome. The objective of this study was to compare mortality in patients suspected of myocardial infarction (MI) presenting with dyspnea versus chest pain in the ambulance.

**Methods:**

Follow-up study in patients undergoing electrocardiogram-based telemedical triage because of suspected MI in an ambulance in the Central Denmark Region from 1 June 2008 to 1 January 2013. Primary outcome was 30-day mortality. Secondary outcomes were 4-year mortality and mortality rates in subgroups of patients with and without a confirmed MI. Absolute risk differences adjusted for comorbidity, age, systolic blood pressure and heart rate were calculated by a generalized linear regression model.

**Results:**

Of 17,398 patients, 12,230 (70 %) suffered from chest pain, 1464 (8 %) from dyspnea, 3540 (20 %) from other symptoms and 164 (1 %) from cardiac arrest. Among patients with dyspnea, 30-day mortality was 13 % (CI 12–15) and 4-year mortality was 50 % (CI 47–54) compared to 2.9 % (CI 2.6-3.2) and 20 % (CI 19–21) in patients with chest pain. MI was confirmed in 121 (8.3 %) patients with dyspnea and in 2319 (19 %) with chest pain. Patients with dyspnea and confirmed MI had a 30-day and 4-year mortality of 21 % (CI 15–30) and 60 % (CI 50–70) compared to 5.0 % (CI 4.2-5.8) and 23 % (CI 21–25) in patients with chest pain and confirmed MI. Adjusting for age, comorbidity, systolic blood pressure and heart rate did not change these patterns.

**Conclusion:**

Patients suspected of MI presenting with dyspnea have significantly higher short- and long-term mortality than patients with chest pain irrespective of a confirmed MI diagnosis. Future studies should examine if supplementary prehospital diagnostics can improve triage, facilitate early therapy and improve outcome in patients presenting with dyspnea.

**Electronic supplementary material:**

The online version of this article (doi:10.1186/s13049-016-0204-9) contains supplementary material, which is available to authorized users.

## Background

In emergency medical services (EMS), a systematic approach to patients suffering high-risk symptoms is essential. Systematic guidelines for prehospital management of chest pain were developed more than a decade ago and prehospital management plays an increasingly important role in cardiac arrest guidelines [1, 2]. Timely prehospital diagnostics and early primary percutaneous coronary intervention or fibrinolysis in ST-elevation myocardial infarction (STEMI) have contributed significantly to a reduced mortality in myocardial infarction (MI) [3, 4]. In out-of-hospital cardiac arrest (OHCA), a combination of layperson cardiopulmonary resuscitation, early defibrillation, improved skills among EMS personnel and advanced life support has improved outcome [5]. For patients with dyspnea, however, we have no commonly accepted guidelines for prehospital management. This is a paradox, since dyspnea is recognized as an independent predictor of mortality [6, 7]. In Denmark, a prehospital electrocardiogram (ECG) based telemedical diagnosis of STEMI is sought not only in patients with chest pain but also among other symptoms suggestive of MI, including dyspnea [4, 8]. However, STEMI is less frequent in patients with dyspnea than in chest pain [9]. In dyspnea, multiple other tentative diagnoses are possible – often as interacting comorbid conditions [10–12]. At the same time, treatment is often necessary before a final diagnosis is made and giving the right treatment to the right patient is a difficult task. We therefore hypothesize that a primary complaint of dyspnea predicts a higher mortality than chest pain in patients suspected of MI in the ambulance.

The primary aim of the present study was to compare mortality in telemedically triaged patients suspected of MI presenting with dyspnea or chest pain. The secondary aim was to compare mortality in these patients according to whether a diagnosis of MI was established or not.

## Methods

### Design and setting

This is a population-based follow-up study of 23,184 telemedical contacts registered in the Danish Tele-database from 1 June 2008 to 1 January 2013. The setting is the Central Denmark Region which covers an area of 13,053 km^2^ of mixed urban, suburban and rural land with 1.3 million inhabitants corresponding to 23 % of the Danish population [13]. The EMS response in this region is two-tiered with basic-level care delivered by ambulances sent to all urgent cases. Anesthesiologist or nurse anesthetist staffed prehospital critical care teams deliver supplementary advanced prehospital care in suspected life-threatening cases. Acutely ill patients are brought to one of five public hospitals within the region. Revascularization therapy (percutaneous coronary intervention or surgery) is centralized at Aarhus University Hospital. Health care is public and provided free of charge by the Danish National Health Service.

Ambulance personnel establish an ECG-based telemedical contact to an on-call cardiologist at a local department of cardiology in patients suspected of an MI according to predefined criteria: 1) patients with on-going or recent chest pain within the past 12 h and/or clinical suspicion of MI (e.g. pallor and diaphoresis) 2) patients with new onset of dyspnea within the past 12 h and no known lung disease 3) patients with other symptoms raising suspicion of heart disease (e.g. syncope/somnolence, palpitations, abdominal/back pain, intoxication etc.) and 4) patients resuscitated from OHCA. In case of STEMI, OHCA with no obvious non-cardiac cause, or cardiogenic shock, the ambulance is re-routed directly to the pre-alerted catheterization laboratory at Aarhus University Hospital for coronary angiography and primary percutaneous coronary intervention. Patients not re-routed directly to the catheterization laboratory are generally admitted to the nearest hospital receiving acute patients. On-call cardiologists receiving the telemedical calls perform all registrations in the Danish Tele-database, including which of the above-mentioned symptoms/conditions is the predominant cause of MI suspicion in the individual patient.

### Study population

The criterion for inclusion in the study was a registration of an ECG-based telemedical contact in the Tele-database at a department of cardiology in the Central Denmark Region with a valid personal identification number. Only the first contact during the study period in each patient was included. Participants were followed until 9 October 2013. Contacts with invalid personal identification number or foreigners were considered lost to follow up and were excluded from the study.

### Data sources

Vital status was retrieved from the Danish Civil Registration System; and data on previous diseases and present diagnoses according to the 10^th^ version of the International Classification of Disease (ICD-10) were retrieved from the Danish National Registry of Patients. Full medical files were retrieved from a total of 200 patients with dyspnea for development of an algorithm for determining the cause of dyspnea in the ambulance and for validation of this algorithm.

The present study was exempt from ethical approval according to Danish law. The Danish Data Protection agency (Journal nr. 1-16-02-158-12) and the Danish Health and Medicines Authority (Journal nr. 3-3013-247/1/) approved the study.

### Exposures and outcomes

The predominant symptoms/conditions leading to suspicion of MI as registered by the on-call cardiologists were regarded as exposures. The primary outcome was 30-day mortality. Secondary outcomes were long-term mortality (4 years), the number of patients in each group diagnosed with an MI and the associated mortality rates, and causes of dyspnea in patients triaged because of dyspnea. The following potential confounders were considered to be relevant: comorbidities, age, gender, smoking status, alcohol consumption, heart rate and systolic blood pressure. Because of an expected mixture of different underlying diseases causing the different symptoms, we opted on using a comorbidity index rather than adjusting for specific diseases. We chose the Charlson Comorbidity Index based on the ICD-10 diagnoses as described by Sundararajan et al. as this is extensively validated  - also in a Danish population [14, 15]. Information on age, gender, heart rate and systolic blood pressure was obtained from the Tele-database. Vital signs were measured at first encounter – in patients resuscitated from cardiac arrest immediately following ROSC. We had no information on smoking status or alcohol consumption.

Patients diagnosed with MI were identified by ICD-10 codes ranging from DI21.0 to DI23.8A. By studying information from the full medical files from the first 100 of the 1,464 patients with dyspnea, we developed an algorithm to predict the cause of dyspnea (heart disease/lung disease/other cause) based on the ICD-10 codes assigned at discharge (Additional file 1 and 2). A new random sample of 100 patients from the 1464 patients with dyspnea was then drawn to test the algorithm. For these patients, full medical files including imaging and blood tests were retrieved and reviewed by an end-point adjudication committee consisting of two investigators blinded to the result of the ICD-10 algorithm. The members of the committee adjudicated the cause of dyspnea, dividing it into one of three main categories: 1) heart disease 2) lung disease and 3) other cause. In case of incongruence, a third investigator reviewed the material and chose one of the two previously adjudicated causes. Thus, the majority vote was accepted as consensus. It was decided a priori that the performance of the ICD-10 algorithm was satisfactory if in agreement with the adjudication committee in at least 75 % of cases. This decision was based on previously reported variations in sensitivity and specificity in which ICD-10 codes were used to evaluate whether a diagnosis of heart failure and chronic obstructive pulmonary disease was established [16, 17].

### Analyses

Mortality was displayed as Kaplan Meier curves which were truncated when less than 10 % of the original cohort was left. To adjust for potential confounders, we planned to perform a Cox proportional hazards regression analysis. The survival curves did not fulfill the proportional hazards assumption, so this plan was waived. Instead we calculated adjusted risk differences by a generalized linear regression using pseudo-observations. The pseudo-observations approach is a transformation of data like log-transformation and others – although it is mathematically more complex. In contrast to Cox regression it allows for non-proportional survival curves and analysis of risk differences at specific time points. It has been well described by Klein et al. [18]. Analyses were conducted in STATA12 (StataCorp LP, Texas, USA) according to the method described by Parner and Andersen [19]. *P*-values <0.05 was considered to be significant and 95 % confidence intervals (CIs) were used. Participants were categorized into Charlson Comorbidity Index Groups according to the original classification by Charlson et al. [20]. Participants were categorized according to systolic blood pressure and heart rate into four groups approximated to quartiles. Missing data were expected to be missing completely at random and no imputation was made.

## Results

**Fig. 1:**
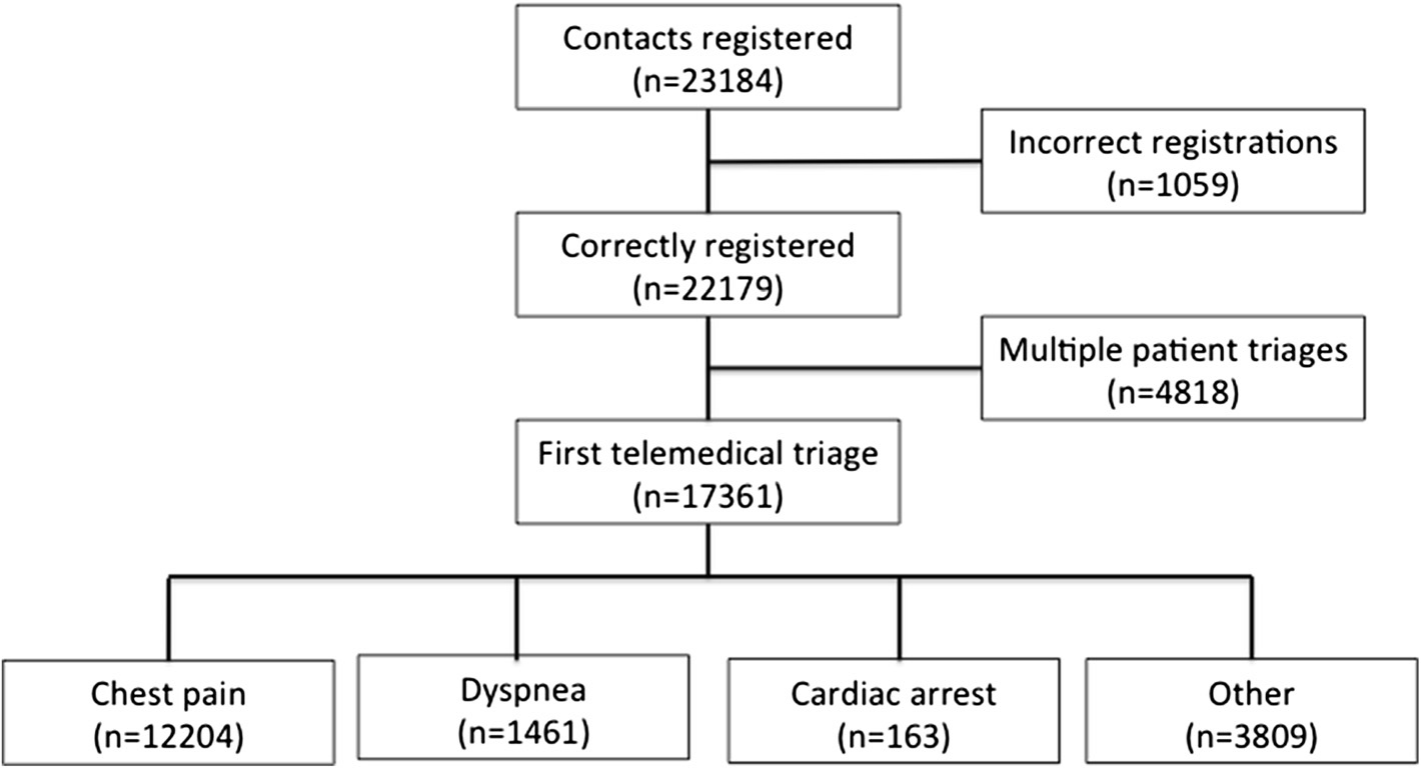
Study flow diagram of patients suspected of myocardial infarction triaged by use of electrocardiogram-based telemedicine

**Fig. 2:**
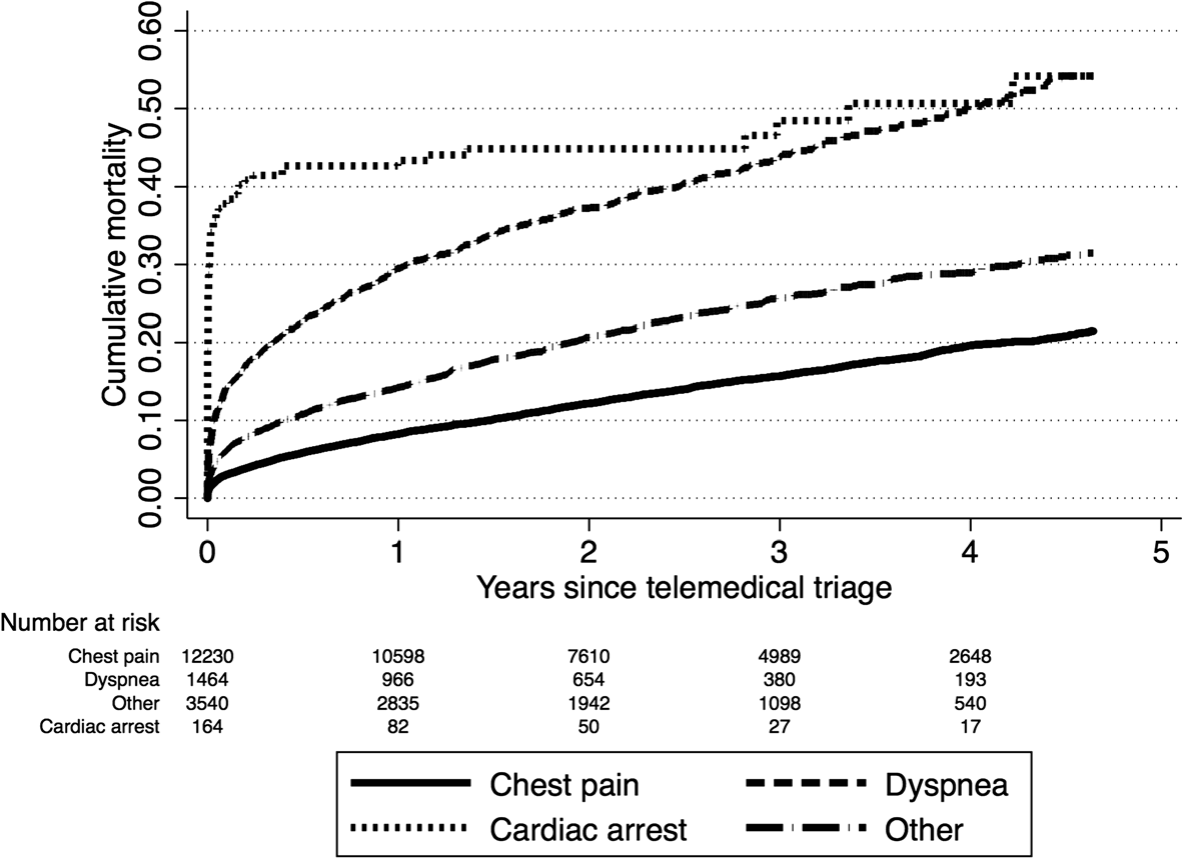
Kaplan Meier curves of cumulative mortality in patients suspected of myocardial infarction triaged by use of electrocardiogram-based telemedicine (*n* = 17,398). Stratified according to symptom/condition leading to telemedical triage

**Fig. 3:**
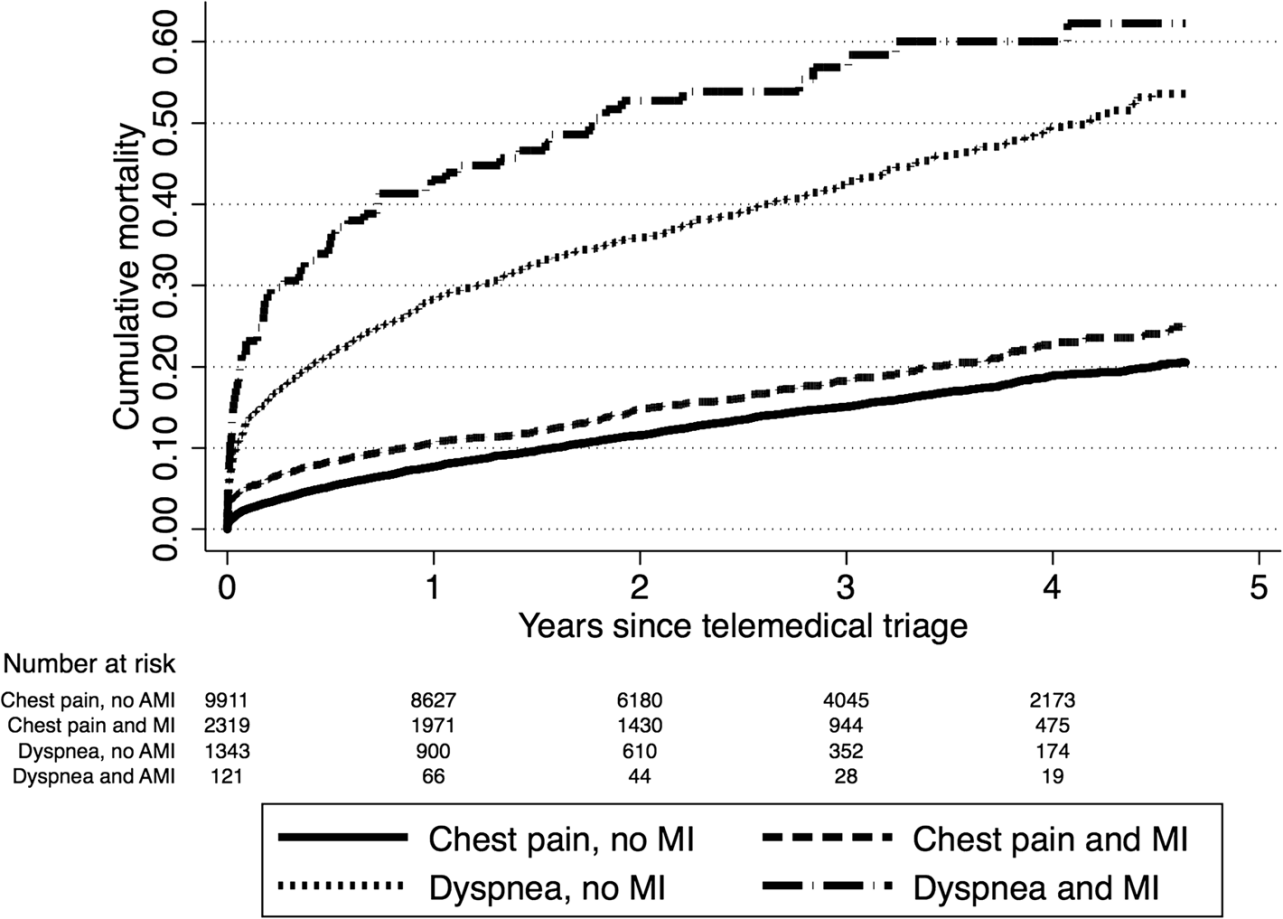
Kaplan Meier curves of cumulative mortality in patients with dyspnea and chest pain suspected of myocardial infarction and triaged by use of electrocardiogram-based telemedicine (*n* = 13,694). Stratified according to whether a myocardial infarction was diagnosed or not

Of 23,184 telemedical contacts, 17,398 correct registrations of first telemedical contacts in individual patients were included (Fig. 1). The patients’ baseline characteristics are presented in Table 1. Median follow-up time was 2.4 years (IQR: 1.3 – 3.7). An MI was diagnosed in 2319 of 12,230 (19 %) patients with chest pain and in 121 of 1464 (8.3 %) patients with dyspnea. Patients suffering dyspnea had higher mortality rates than patients with chest pain at both day 30 (primary endpoint) and 4 years, irrespective of whether a diagnosis of MI was confirmed or not (Table 2). Kaplan-Meier estimates for mortality in all four groups of patients are displayed in Fig. 2. Kaplan-Meier estimates for mortality in patients with chest pain and dyspnea with and without confirmed MI are displayed in Fig. 3. Age, comorbidity, systolic blood pressure and heart rate remained significant predictors of mortality in the generalized linear regression model. Adjusted for these potential confounders, patients suffering dyspnea still had higher mortality rates than patients suffering chest pain (Table 3).

**Table 1:** Baseline characteristics for patients suspected of myocardial infarction triaged by use of electrocardiogram-based telemedicine (*n* = 17,398). Stratified according to symptom/condition leading to telemedical triage

	Chest pain		Dyspnea		Cardiac arrest		Other		
	(*n* = 12,230)		(*n* = 1464)		(*n* = 164)		(*n* = 3540)		
Variables	No (%)	Valid cases	No (%)	Valid cases	No (%)	Valid cases	No (%)	Valid cases	*p*
Demographics									
Age, median (IQR), y	65 (53–76)	12,230	76 (65–84)	1464	67 (57–79)	163	71 (59–81)	3540	<0.001
Sex (male)	7061 (58)	12,230	760 (52)	1464	114 (70)	163	2098 (59)	3540	<0.001
Charlson comorbidity index group		12,230		1464		163		3540	
0	6296 (51)		483 (33)		90 (55)		1638 (46)		<0.001
1–2	4016 (33)		517 (35)		57 (35)		1235 (35)		<0.001
3–4	1274 (10)		279 (19)		11 (7)		441 (12)		<0.001
≥5	644 (5)		185 (13)		6 (4)		226 (6)		<0.001
Clinical parameters									
Blood pressure									
Systolic, median (IQR), mmHg	146 (129–166)	11,449	148 (128–170)	1362	129 (107–148)	124	138 (120–158)	3240	<0.001
Diastolic, median (IQR), mmHg	88 (77–99)	11,411	87 (74–102)	1361	83 (66–102)	123	82 (70–94)	3231	<0.001
Heart Rate, median (IQR), beats/min	82 (70–98)	11,451	95 (80–116)	1373	92 (76–105)	127	82 (67–101)	3242	<0.001

Binary data are compared using chi-squared test, continuous data are compared using Kruskal Wallis test

**Table 2:** Crude mortality rates in patients suspected of myocardial infarction triaged by use of electrocardiogram-based telemedicine (*n* = 17,398). Stratified according to symptom/condition leading to telemedical triage and to myocardial infarction diagnosis in patients with dyspnea and chest pain

	Chest pain (*n* = 12,230)			Dyspnea (*n* = 1464)			Cardiac arrest (*n* = 164)	Other (*n* = 3540)
	Overall	No MI	MI	Overall	No MI	MI		
Mortality	(*n* = 12,230)	(*n* = 9911)	(*n* = 2319)	(*n* = 1464)	(*n* = 1343)	(*n* = 121)		
30 days (95% CI)	2.9 % (2.6–3.2)	2.3 % (2.1–2.7)	5.0 % (4.2–5.8)	13 % (12–15)	13 % (11–15)	21 % (15–30)	38 % (31–46)	5.8 % (5.0–6.6)
4 years 95% CI	20 % (19–21)	19 % (18–20)	23 % (21–25)	50 % (47–54)	50 % (46–53)	60 % (50–70)	51 % (42–60)	29 % (27–31)

Abbreviations: *MI* myocardial infarction

**Table 3:** Generalized linear regression analysis of covariates associated with mortality. Analyzed at 30 days and 4 years in patients suspected of myocardial infarction triaged by use of electrocardiogram-based telemedicine (*n* = 15,578 cases with complete data)

	30-day mortality		4-year mortality	
Covariates remaining significant in models				
	Risk difference (95 % CI)	*P* value	Risk difference (95 % CI)	*P* value
Demographics				
Age, per 1-y increase	0.19 % (0.17 to 0.21)	<0.001	0.9 % (0.8 to 1.0))	<0.001
Comorbidity				
Charlson Comorbidity Index				
0	1 [Reference]		1 [Reference]	
1–2	0.1 % (−0.6 to 0.8)	0.852	7.2 % (5.4 to 8.9)	<0.001
3–4	1.6 % (0.3 to 2.8)	0.017	22 % (19 to 26)	<0.001
≥5	6.4 % (4.3 to 8.6)	<0.001	39 % (35 to 44)	<0.001
Clinical characteristics				
Systolic blood pressure				
<120	1 [Reference]		1 [Reference]	
120–139	−4.9 % (−6.2 to −3.6)	<0.001	−3.7 % (−6.2 to −1.2)	0.004
140–159	−6.0 % (−7.3 to −4.8)	<0.001	−7.3 % (−9.9 to −4.8)	<0.001
≥160	−7.8 % (−9.1 to −6.5)	<0.001	−11 % (−13 to −8.0)	<0.001
Heart rate				
<70	1 [Reference]		1 [Reference]	
70–84	1.3 % (0.5 to 2.1)	0.001	4.1 % (2.0 to 6.3)	<0.001
85–99	2.2 % (1.3 to 3.1)	<0.001	5.2 % (2.9 to 7.5)	<0.001
≥100	3.2 % (2.2 to 4.1)	<0.001	8.7 % (6.5 to 10.9)	<0.001
Patient category				
Chest pain, no MI	1 [Reference]		1 [Reference]	
Chest pain and MI	1.8 % (0.8 to 2.8)	<0.001	1.7 % (−0.7 to 4.0)	0.167
Dyspnea, no MI	7.3 % (5.5 to 9.2)	<0.001	16 % (12 to 19)	<0.001
Dyspnea and MI	12 % (5.7 to 20)	<0.001	21 % (11 to 32)	<0.001
Other	1.7 % (0.8 to 2.6)	<0.001	3.8 % (1.7 to 5.8)	<0.001
Cardiac arrest	26 % (18 to 34)	<0.001	20 % (11 to 29)	<0.001

Abbreviations: *MI* myocardial infarction

### Cause of dyspnea

Two endpoint committee adjudicators agreed on the primary cause of dyspnea in all but four of 100 patients sampled for validation. In these patients, the third adjudicator was involved and the majority vote was accepted. The ICD-10 diagnosis algorithm was in agreement with the endpoint adjudication committee in 78 % of the 100 patients sampled for validation. The predominant underlying cause of dyspnea in this cohort of patients suspected of MI was heart disease, but only 8,3 % actually had an MI (Table 4). Among patients in whom heart disease was the primary cause of dyspnea, 20 % were also diagnosed with a lung disease. When lung disease was the primary cause for dyspnea, 41 % of patients were also diagnosed with a heart disease.

**Table 4:** Diagnoses in patients with dyspnea. Primary diagnoses in patients suspected of myocardial infarction because of dyspnea and triaged by use of electrocardiogram-based telemedicine (*n* = 1464)

Primary diagnosis	*n* (%)
Heart disease	692 (47.3)
Heart failure/cardiomyopathies	202 (13.8)
Supraventricular tachycardia	151 (10.3)
Acute myocardial infarction	121 (8.3)
Pulmonary embolism	64 (4.4)
Chronic ischaemic heart disease	57 (4.0)
Valvular heart disease	46 (3.2)
Other arrhythmia	17 (1.2)
Other heart disease	34 (2.3)
Lung disease	359 (24.5)
Pulmonary infections	209 (14.3)
Obstructive lung diseases	121 (8.3)
Pleural disorders	12 (0.8)
Interstitial lung disease	8 (0.6)
Other lung diseases	9 (0.6)
Other	413 (28.2)
Other infections	49 (3.4)
Gastrointestinal diseases	31 (2.1)
Endocrine and metabolic diseases	26 (1.8)
Anaemia	18 (1.2)
Renal disease	18 (1.2)
Miscellaneous	133 (9.1)
No final diagnosis	138 (9.4)
Symptom/“encounter for” diagnoses	105 (7.1)
No hospital admission	33 (2.3)

### Sensitivity analyses

We performed three sensitivity analyses, all related to different ways of adjusting for age and comorbidity (included in Additional file 3). We accounted for a possible higher risk with increasing age in older age categories than in younger by including both age and age squared and for a possible polynomial development by including age as qubic splines with seven knots. This reduced the effect of dyspnea slightly but did not change the overall patterns. Including specific diseases related to chest pain and dyspnea rather than the Charlson Comorbidity Index in addition to using qubic splines to adjust for age did not change the results.

## Discussion

This study demonstrates that among patients suspected of MI in the ambulance, patients presenting dyspnea have a three-fold higher mortality than patients with chest pain irrespective of a confirmation of the MI diagnosis. Adjustment for potential confounders does not change these patterns.

### Dyspnea vs. other symptoms

Studies comparing mortality rates in patients with dyspnea and patients with other symptoms in a prehospital setting are scarce. However, a large study in emergency medical technician witnessed OHCA showed that patients suffering chest pain prior to cardiac arrest were five times more likely to survive than patients suffering dyspnea prior to cardiac arrest [21]. In a retrospective analysis of hospitalized patients with first-time MI, dyspnea was associated with higher long-term mortality than other presenting symptoms, including chest pain [22]. In patients undergoing coronary angiography for suspected acute coronary syndrome, a four-fold higher mortality in dyspneic compared to non-dyspneic patients was demonstrated [23]. Thus, the observation of higher mortality among dyspneic patients is consistent across the prehospital setting, the acute hospital setting, and the catheterization laboratory. Nevertheless, we still do not have broadly accepted guidelines for management of the dyspneic patient in the prehospital setting.

### Diagnoses, comorbidities and age

Approximately 8 % of the patients suffering dyspnea had an MI. ECG acquisition is thus important also in prehospital diagnostics in dyspnea patients, but it seems inadequate to establish a prehospital diagnosis and identify high-risk patients in this patient group in general. These patients suffer from a variety of diseases not only including MI but also heart failure, pulmonary infections, and obstructive pulmonary disorders. Furthermore, our study also demonstrates that heart and lung disease frequently coexist. This is supported by other studies [11, 12], and this co-existence deteriorates the prognosis [10]. As it is seen from Table 1, age and comorbidities do play a major role in the high mortality in patients with dyspnea. At long term, the independent effect on mortality of being in CCI group ≥ 3 or being 10 years older is more pronounced than the effect of suffering dyspnea. At short term, the same applies to age, but not comorbidities. In addition, no matter how we tried to adjust for age and comorbidities, we were not able to eliminate an independent effect of dyspnea compared to chest pain. This indicates, that there is a potential for improving care of patients with dyspnea. Based on the very high mortality observed in the first short period of time after ambulance contact, there seems to be a window of opportunity in the acute phase (refer to the Kaplan-Meier curves in Fig. 2).

### Towards a systematic approach

As of now, a stethoscope and an ECG is the most advanced diagnostics routinely used in dyspneic patients in the prehospital setting. Treatment is often broad rather than specific and the evidence behind different new interventions is scarce. We are currently witnessing several, promising technological developments that may aid prehospital decision-making and treatment in dyspneic patients. Point-of-care testing with biomarkers, like brain natriuretic peptides and focused ultrasonography, has been suggested [24–26]. Establishing an accurate diagnosis already in the ambulance to facilitate earlier correct therapy and triage high-risk patients to specialized departments or intensive care unit may improve outcome, but this has not been examined. New treatment regimes facilitating oxygenation and ventilation by continuous positive airway pressure or non-invasive ventilation has also been suggested, but has not been sufficiently examined in relation to patient outcome [27]. Validating the findings of this study in unselected patients with dyspnea in the prehospital setting and the systematic use of prehospital point-of-care diagnostics and novel treatments for this patient group are potential subjects for future research.

### Strengths and limitations

A high number of consecutively registered patients and adjustment for major confounding factors including age and comorbidity are strengths of this study. Unlike most epidemiological studies, we were also able to adjust for prehospital values of systolic blood pressure and heart rate. An endpoint-adjudication committee validated the use of ICD-10 diagnoses to establish the cause of dyspnea. Owing to the predefined criteria for telemedical contact, only a fraction of all patients with dyspnea in the ambulance were telemedically triaged and registered. A selection bias that favors a higher proportion of patients with heart disease is evident. Dyspneic patients suspected of MI might be more severely affected than those not suspected of MI because some of them may have large anterior infarctions and non-cardiac acute conditions like pulmonary embolism and pneumothorax. This might have led to an overestimation of the mortality in patients with dyspnea compared with a general prehospital dyspnea population. As we did not have data on smoking status, alcohol consumption, left ventricular systolic function or pulmonary function, residual confounding might be present. However, adjusting for smoking status did not change the association between dyspnea and mortality in several other studies [7, 28]. Pulmonary disease and heart failure are included in the Charlson Comorbidity Index and adjustment was inherent for pulmonary function and left ventricular systolic function.

## Conclusion

Patients suspected of MI presenting with dyspnea have a markedly higher short- and long-term mortality than patients with chest pain irrespective of the presence of MI. ECG acquisition and interpretation are insufficient to diagnose such high-risk patients in this group. Further studies are needed to clarify whether supplementary prehospital diagnostics can improve triage, facilitate earlier correct therapy and improve outcome in patients presenting with dyspnea.

## Additional files

Additional file 1:
**The ICD-10 diagnosis algorithm.** (PDF 38 kb) Additional file 2:
**ICD-10 codes (text in Danish).** (XLSX 610 kb) Additional file 3:
**Sensitivity analyses of the generalized linear regressions analyses of covariates associated with mortality.** Results of regression analysis using 1) age/age2 2) restricted cubic splines 3) restricted cubic splines and specific diagnosis. Analyzed at 30 days and 4 years in patients suspected of myocardial infarction triaged by use of electrocardiogram-based telemedicine (*n* = 15,578 cases with complete data). (DOCX 102 kb)
