# Supplementary material for: Dyspnea, a high-risk symptom in patients suspected of myocardial infarction in the ambulance? A population-based follow-up study
Source: Scand J Trauma Resusc Emerg Med. 2016 Feb 12;24:15. doi: 10.1186/s13049-016-0204-9 (PMC4751637; doi:10.1186/s13049-016-0204-9)
Supplement: Additional file 1: — The ICD-10 diagnosis algorithm. (PDF 38 kb) [file 13049_2016_204_MOESM1_ESM.pdf]

Additional file 1 - The ICD-10 diagnosis algorithm

1. All ICD10-codes (and ICD codes from previous ICD versions) were divided into four categories – heart, lung, other and symptom diagnoses (categorized ICD-10 codes can be found in appendix 2)
2. The primary diagnosis relating to admission was primarily screened (any prehospital diagnoses were omitted)
    a. If this belonged to one of the categories heart, lung or other, the search was ended and this diagnosis was regarded as final
3. If the primary diagnosis belonged to the category symptom diagnoses, secondary diagnoses were screened
    a. If one or more diagnoses within one and only one of the categories heart, lung and other were identified, the first consecutive diagnosis of these diagnoses was regarded as the final diagnosis
    b. If diagnoses within more than one of these categories were identified, the case was marked for manual selection of diagnoses
        i. The primary investigator reviewed cases marked for manual search and adjudicated a final diagnosis by choosing one of the secondary diagnoses. An example is the combination of I10.9 Hypertensio arterialis essentiales and I48.9B Atrial fibrillation. I489B Atrial Fibrillation was then chosen. Another frequent encounter was the combination J18.9 Pneumonia and I489BB Atrial Fibrillation. J18.9 Pneumonia was then chosen
4. If there were no secondary diagnoses or these belonged only to the category symptom diagnoses, the next consecutive admission (in a line of admission following immediately after each other) was screened according to 2-3. Admissions that started more than 30 days before the index event were omitted
    a. If there were no admissions immediately following the initial one or if diagnoses only belonged to the category symptom diagnoses, the search was ended and the final diagnosis was regarded as symptom or "encounter for…" diagnosis and transferred to the other category
5. This yielded adjudication into three final categories – heart, lung and other.
