# Supplementary material for: Dyspnea, a high-risk symptom in patients suspected of myocardial infarction in the ambulance? A population-based follow-up study
Source: Scand J Trauma Resusc Emerg Med. 2016 Feb 12;24:15. doi: 10.1186/s13049-016-0204-9 (PMC4751637; doi:10.1186/s13049-016-0204-9)
Supplement: Additional file 3: — Sensitivity analyses of the generalized linear regressions analyses of covariates associated with mortality. Results of regression analysis using 1) age/age2 2) restricted cubic splines 3) restricted cubic splines and specific diagnosis. Analyzed at 30 days and 4 years in patients suspected of myocardial infarction triaged by use of electrocardiogram-based telemedicine (n = 15,578 cases with complete data). (DOCX 102 kb) [file 13049_2016_204_MOESM3_ESM.docx]

| **Additional file 3. Sensitivity analyses of the generalized linear regressions analyses of covariates associated with mortality**  **Sensitivity analysis 1. Results of regression analysis using age and age squared to adjust for age**  Analyzed at 30 days and 4 years in patients suspected of myocardial infarction triaged by use of electrocardiogram-based telemedicine (n = 15,578 cases with complete data). | | | | | | |
| --- | --- | --- | --- | --- | --- | --- |
|  | | **30-day mortality** | |  | **4-year mortality** | |
| **Covariates remaining significant in models** | |  |  |  |  |  |
|  |  | Risk difference (95% CI) | P value |  | Risk difference (95% CI) | P value |
| Demographics | |  |  |  |  |  |
| Age, per 1-y increase | | -0.43% (-0.53 to -0.32) | <0.001 |  | -0.22% (-0.24 to -0.20) | <0.001 |
| Age^2^, per 1-step increase | | 0.005% (0.004 to 0.006) | <0.001 |  | 0.026% (0.024 to 0.028) | <0.001 |
| Comorbidity | |  |  |  |  |  |
| Charlson Comorbidity Index | |  |  |  |  |  |
| 0 | | 1 [Reference] |  |  | 1 [Reference] |  |
| 1-2 | | 0.2% (-0.5 to 0.9) | 0.574 |  | 7.8% (6.1 to 9.6) | <0.001 |
| 3-4 | | 1.6% (0.3 to 2.9) | 0.014 |  | 23% (19 to 26) | <0.001 |
| ≥5 | | 6.4% (4.3 to 8.6) | <0.001 |  | 40% (36 to 44) | <0.001 |
| Clinical characteristics | |  |  |  |  |  |
| Systolic blood pressure | |  |  |  |  |  |
| <120 | | 1 [Reference] |  |  | 1 [Reference] |  |
| 120-139 | | -5.0% (-6.3 to -3.7) | <0.001 |  | -4.3% (-6.7 to -1.8) | 0.001 |
| 140-159 | | -5.9% (-7.2 to -4.6) | <0.001 |  | -6.9% (-9.3 to -4.4) | <0.001 |
| ≥160 | | -7.5% (-8.7 to -6.2) | <0.001 |  | -8.9% (-11 to -6.5) | <0.001 |
| Heart rate | |  |  |  |  |  |
| <70 | | 1 [Reference] |  |  | 1 [Reference] |  |
| 70-84 | | 1.3% (0.5 to 2.1) | 0.002 |  | 3.8% (1.7 to 5.9) | <0.001 |
| 85-99 | | 2.0% (1.1 to 2.9) | <0.001 |  | 4.4% (2.2 to 6.7) | <0.001 |
| ≥100 | | 3.1% (2.1 to 4.0) | <0.001 |  | 8.1% (6.9 to 10) | <0.001 |
| Patient category | |  |  |  |  |  |
| Chest pain, no MI | | 1 [Reference] |  |  | 1 [Reference] |  |
| Chest pain and MI | | 2.1% (1.2 to 3.1) | <0.001 |  | 3.1% (0.8 to 5.4) | 0.009 |
| Dyspnea, no MI | | 6.9% (5.1 to 8.8) | <0.001 |  | 14% (10 to 17) | <0.001 |
| Dyspnea and MI | | 13% (5.4 to 20) | <0.001 |  | 17% (7 to 27) | <0.001 |
| Other | | 1.4% (0.8 to 2.3) | <0.001 |  | 2.2% (0.2 to 4.2) | <0.001 |
| Cardiac arrest | | 26% (18 to 35) | <0.001 |  | 21% (13 to 30) | <0.001 |
| Abbreviations: MI, myocardial infarction | | | | | | |

| **Sensitivity analysis 2. Results of regression analysis using restricted cubic splines to adjust for age**  Analyzed at 30 days and 4 years in patients suspected of myocardial infarction triaged by use of electrocardiogram-based telemedicine (n = 15,578 cases with complete data). Knots generated at age = 29, 50 59, 66, 74, 81, 91. | | | | | | |
| --- | --- | --- | --- | --- | --- | --- |
|  | | **30-day mortality** | |  | **4-year mortality** | |
| **Covariates remaining significant in models** | |  |  |  |  |  |
|  |  | Risk difference (95% CI) | P value |  | Risk difference (95% CI) | P value |
| Demographics | |  |  |  |  |  |
| Age spline 1 | | 0.05% (0.008 to 0.09) | 0.018 |  | 0.02% (-0.1 to 0.1) | 0.732 |
| Age spline 2 | | 0.02% (-0.3 to -0.03) | 0.13 |  | 1.0% (0.2 to 1.9) | 0.019 |
| Age spline 3 | | -0.8% (-4.7 to 3.1) | 0.697 |  | -12% (-23 to -1.6) | 0.024 |
| Age spline 4 | | 6.6% (-7.8 to 21) | 0.369 |  | 53% (16 to 89) | 0.004 |
| Age spline 5 | | -12% (-36 to 13) | 0.355 |  | -68% (-126% to -10%) | 0.021 |
| Age spline 6 | | 13% (-17 to 43) | 0.392 |  | 38% (-27 to 104) | 0.253 |
| Comorbidity | |  |  |  |  |  |
| Charlson Comorbidity Index | |  |  |  |  |  |
| 0 | | 1 [Reference] |  |  | 1 [Reference] |  |
| 1-2 | | 0.3% (-0.4 to 1.0) | 0.413 |  | 8.2% (6.4 to 9.9) | <0.001 |
| 3-4 | | 1.8% (0.5 to 3.1) | 0.005 |  | 23% (20 to 27) | <0.001 |
| ≥5 | | 6.9% (4.8 to 9.0) | <0.001 |  | 41% (37 to 45) | <0.001 |
| Clinical characteristics | |  |  |  |  |  |
| Systolic blood pressure | |  |  |  |  |  |
| <120 | | 1 [Reference] |  |  | 1 [Reference] |  |
| 120-139 | | -5.1% (-6.3 to -3.8) | <0.001 |  | -4.6% (-7.0 to -2.1) | <0.001 |
| 140-159 | | -6.0% (-7.2 to -4.7) | <0.001 |  | -7.2% (-9.7 to -4.7) | <0.001 |
| ≥160 | | -7.5% (-8.8 to -6.2) | <0.001 |  | -8.9% (-11 to -6.5) | <0.001 |
| Heart rate | |  |  |  |  |  |
| <70 | | 1 [Reference] |  |  | 1 [Reference] |  |
| 70-84 | | 1.2% (0.5 to 2.0) | 0.002 |  | 3.7% (1.7 to 5.8) | <0.001 |
| 85-99 | | 2.0% (1.1 to 2.9) | <0.001 |  | 4.3% (2.1 to 6.6) | <0.001 |
| ≥100 | | 3.1% (2.1 to 4.0) | <0.001 |  | 8.0% (5.8 to 10) | <0.001 |
| Patient category | |  |  |  |  |  |
| Chest pain, no MI | | 1 [Reference] |  |  | 1 [Reference] |  |
| Chest pain and MI | | 2.2% (1.2 to 3.1) | <0.001 |  | 3.3% (0.9 to 6.5) | 0.006 |
| Dyspnea, no MI | | 6.9% (5.1 to 8.8) | <0.001 |  | 14% (10 to 17) | <0.001 |
| Dyspnea and MI | | 12% (5.4 to 20) | <0.001 |  | 17% (7 to 27) | 0.001 |
| Other | | 1.5% (0.7 to 2.4) | <0.001 |  | 2.8% (0.8 to 4.8) | <0.001 |
| Cardiac arrest | | 26% (18 to 35) | <0.001 |  | 22% (13 to 30) | 0.006 |
| Abbreviations: MI, myocardial infarction | | | | | | |

| **Sensitivity analysis 3. Results of regression analysis using restricted cubic splines to adjust for age and specific diagnoses to adjust for comorbidity**  Analyzed at 30 days and 4 years in patients suspected of myocardial infarction triaged by use of electrocardiogram-based telemedicine (n = 15,578 cases with complete data). Knots generated at age = 29, 50 59, 66, 74, 81, 91. | | | | | | |
| --- | --- | --- | --- | --- | --- | --- |
|  | | **30-day mortality** | |  | **4-year mortality** | |
| **Covariates remaining significant in models** | |  |  |  |  |  |
|  |  | Risk difference (95% CI) | P value |  | Risk difference (95% CI) | P value |
| Demographics | |  |  |  |  |  |
| Age spline 1 | | 0.05% (-0.01 to 0.10) | 0.014 |  | 0.06% (-0.06 to 0.18) | 0.318 |
| Age spline 2 | | 0.02% (-0.3 to 0.3) | 0.904 |  | 1.0% (0.2 to 1.9) | 0.021 |
| Age spline 3 | | -0.6% (-4.6 to 3.3) | 0.752 |  | -11% (-22 to -0.3) | 0.044 |
| Age spline 4 | | 6.0% (-8.5 to 20) | 0.419 |  | 46% (10 to 83) | 0.012 |
| Age spline 5 | | -11% (-35 to 14) | 0.400 |  | -58% (-116 to -0.2) | 0.049 |
| Age spline 6 | | 12% (-18 to 42) | 0.441 |  | 27% (-39 to 93) | 0.424 |
| Comorbidity | |  |  |  |  |  |
| Specific diagnoses | |  |  |  |  |  |
| Prior MI | | -0.6 (-1.7 to 0.5) | 0.299 |  | 2.4 (-0.5 to 5.4) | 0.099 |
| Heart failure | | 2.4% (1.0 to 3.8) | 0.001 |  | 15% (12 to 19) | <0.001 |
| Chronic pulmonary disorder | | 1.1% (-0.1 to 2.3) | 0.070 |  | 14% (11 to 17) | <0.001 |
| Chronic renal failure | | 4.7% (2.3 – 7.1) | <0.001 |  | 21% (16 to 26) | <0.001 |
| Hypertension | | 0.1 (-0.7 to 1.0) | 0.739 |  | 0.6% (-1.4 to 2.7) | 0.541 |
| Diabetes | | 0.6% (-0.6 to 1.8) | 0.344 |  | 8.0 (5.0 to 11) | <0.001 |
| Clinical characteristics | |  |  |  |  |  |
| Systolic blood pressure | |  |  |  |  |  |
| <120 | | 1 [Reference] |  |  | 1 [Reference] |  |
| 120-139 | | -5.0% (-6.9 to -3.7) | <0.001 |  | -4.7% (-7.3 to -2.3) | <0.001 |
| 140-159 | | -6.0% (-7.2 to -4.7) | <0.001 |  | -7.4% (-9.9 to -4.9) | <0.001 |
| ≥160 | | -7.5% (-8.8 to -6.2) | <0.001 |  | -9.4% (-12 to -6.8) | <0.001 |
| Heart rate | |  |  |  |  |  |
| <70 | | 1 [Reference] |  |  | 1 [Reference] |  |
| 70-84 | | 1.2% (0.5 to 2.0) | 0.002 |  | 3.8% (1.8 to 5.9) | <0.001 |
| 85-99 | | 2.0% (1.1 to 2.9) | <0.001 |  | 4.4% (2.2 to 6.7) | <0.001 |
| ≥100 | | 3.2% (2.2 to 4.1) | <0.001 |  | 8.6% (6.5 to 11) | <0.001 |
| Patient category | |  |  |  |  |  |
| Chest pain, no MI | | 1 [Reference] |  |  | 1 [Reference] |  |
| Chest pain and MI | | 2.2% (1.3 to 3.2) | <0.001 |  | 3.3% (1.0 to 5.6) | 0.006 |
| Dyspnea, no MI | | 6.9% (5.1 to 8.9) | <0.001 |  | 13% (9.5 to 17) | <0.001 |
| Dyspnea and MI | | 12% (5.3 to 19) | <0.001 |  | 16% (6 to 26) | 0.001 |
| Other | | 1.5% (0.6 to 2.3) | <0.001 |  | 2.6% (0.5 to 4.6) | <0.001 |
| Cardiac arrest | | 26% (18 to 34) | <0.001 |  | 20% (12 to 29) | 0.013 |
| Abbreviations: MI, myocardial infarction | | | | | | |
